# Don’t Get Me Wrong: ERP Evidence from Cueing Communicative Intentions

**DOI:** 10.3389/fpsyg.2017.01465

**Published:** 2017-09-11

**Authors:** Stefanie Regel, Thomas C. Gunter

**Affiliations:** ^1^Department of Neuropsychology, Max Planck Institute for Human Cognitive and Brain Sciences Leipzig, Germany; ^2^Department of Neurocognitive Psychology, Humboldt University of Berlin Berlin, Germany

**Keywords:** ERP, P600, N400, language comprehension, social cognition, cueing, pragmatics, irony

## Abstract

How to make sure that one’s utterances are understood as intended when not facing each other? In order to convey communicative intentions, in digital communication emoticons and pragmatic cues are frequently used. Such cueing becomes even more crucial for implied interpretations (e.g., irony) that cannot be understood literally, but require extra information. Sentences, such as ‘*That’s fantastic*,’ may achieve either a literal or ironic meaning depending on the contextual constraints. In two experiments using event-related brain potentials (ERPs), we examined the effects of cueing communicative intentions (i.e., by means of quotation marks) on ironic and literal language comprehension. An impact of cueing on language processing was seen as early as 200 ms post-stimulus onset by the emergence of a P300 preceding a sustained positivity for cued irony relative to literal language, while for uncued irony a P200-P600 pattern was obtained. In presence of additional information for ironic intentions, pragmatic reanalysis allowing inferences on the message level may have occured immediately. Moreover, by examining the way of cueing (i.e., ambiguous vs. unambiguous cueing) this type of information for communicative intentions appeared to be only effective when the cues were unambiguous by matching pragmatic conventions. The findings suggest that cueing communicative intentions may immediately affect language comprehension, albeit depending on pragmatic conventions of the cues’ usage.

## Introduction

Face-to-face communication allows to convey the speaker’s emotions and attitudes in various ways by means of speech prosody, facial expressions, or gestures besides to language use. In digital communication via posts, email and text messages, however, a speaker primarily depends on verbal information. The missing prosodic, facial and gestural cues are commonly substituted by communicative cues including emoticons and various types of pragmatic cues for conveying the intended meanings (see e.g., Dresner and Herring, 2010; Vandergriff, 2013). By means of graphic signs representing objects or facial expressions, and punctuation (e.g., repeatedly occurring exclamation and question marks) co-occurring with the verbal message additional information for the speaker’s intention is provided. Among punctuation, quotation marks serve as minimal pragmatic markers signalizing an alternative interpretation of the words and phrases put in quotes (Gutzmann and Stei, 2011). Functioning as an attention getting device is inherent in all cues for inducing enhanced salience of the cued information. Cueing communicative intentions becomes even more important when dealing with implied interpretations that cannot be derived from verbal information. When joking or using non-literal language, such as metaphors or irony, contextual information and pragmatic knowledge (i.e., speakers’ mutual knowledge about language use) is crucial for inferring the speaker’s intention. Imagine, for example, two friends with one of them promising to buy tickets for a visit to a concert, but finally forgot to do so. When telling to his friend, she might reply by saying *That’s “fantastic”* expressing her disappointment ironically. In order to avoid misinterpretation, the use of quotation and exclamations marks may enrich the speaker’s message. In the ironic example above, the intended message entails some degree of opposite meaning of the literal sentence interpretation, with quotes emphasizing this deviation in meaning. Understanding non-literal meanings requires the integration of verbal (e.g., semantic, syntactic, and pragmatic information) and contextual information. According to Friederici (2002), sentence comprehension involves initial syntactic structure building based on word category information followed by lexical-semantic and morphosyntactic processes. During a final stage of language processing, these different types of information are integrated into complete sentence representations. With respect to non-literal language comprehension, the cognitive processes engaged in deriving implied interpretations are differentially modeled. The *standard pragmatic view* (based on the work of Grice, 1975) assumes that literal sentence meanings are initially accessed resulting in semantic integration difficulty during integration of verbal with contextual information. Inferential processes are necessitated to derive contextually appropriate meanings. In contrast, the *direct access model* (Gibbs, 2002) assumes that lexical-semantic and contextual information interacts from early on allowing an immediate access of contextually relevant meanings and thus a direct understanding of the implied interpretations. Recent neurocognitive evidence favors an engagement of pragmatic reanalysis (presumably comprising inferential processes) when comprehending irony (Regel et al., 2010, 2011; Spotorno et al., 2013). Semantic integration difficulty, however, not necessarily occurs suggesting an adaption of the *standard pragmatic view*: Comprehending irony engages later inferential processes but no enhanced lexical-semantic integration. In neuroimaging research an extended network of brain activations comprising the left inferior frontal gyrus and bilateral inferior temporal cortex was seen for metaphors, while for irony the right superior and middle temporal gyri were activated (Eviatar and Just, 2006). In order to image the temporal processing underlying language comprehension ERPs allow time-sensitive measurements of the brain activity. The observation of an N400 component peaking around 400 ms post-stimulus onset has primarily been associated with lexical-semantic processes (for review see Kutas and Federmeier, 2011). The P600 component (i.e., a late positivity emerging around 500 ms post-stimulus) has been obtained for syntactic (for review see Gouvea et al., 2010) as well as semantic-pragmatic information processing (e.g., Brouwer et al., 2012; Regel et al., 2014). An additional language-related ERP component, the P200, with a latency onset of 200 ms post-stimulus presentation was seen for semantic relatedness between word pairs (Landi and Perfetti, 2007), for highly vs. weakly constrained sentence completions (Federmeier et al., 2005; Wlotko and Federmeier, 2007), as well as for non-literal sentence endings (Regel et al., 2011; Spotorno et al., 2013; Schneider et al., 2014). The P200 thus may be sensitive to early semantic analysis processes of words presented in sentential contexts. In case of non-literal meanings words’ semantic features biased by contextual information may be analyzed and associated with the contexts. Previous ERP studies on irony comprehension have revealed a reliable P200–P600 pattern for irony relative to literal language in absence of an enhanced N400 (Regel et al., 2010, 2011; Spotorno et al., 2013) suggesting that semantic integration (indexed by N400) was comparable for both literal and ironic meanings, presumably due to earlier semantic processes (indicated by P200). During later stages of processing pragmatic reanalysis (reflected by P600) was involved for deriving appropriate non-literal meanings. In comparison, metaphors seem to engage more extended semantic-conceptual integration as shown by an N400-P600 pattern (e.g., Coulson and Van Petten, 2002), which accords with findings of enhanced activation in the middle temporal cortex in response to metaphors (Eviatar and Just, 2006), one of the regions generating N400 (for review see Lau et al., 2008). An engagement of different processing mechanisms for metaphors and sarcasm was also confirmed in visual field studies, with sarcasm showing greater activation of the right hemisphere (Briner et al., 2011). Other studies revealed modulations of N400 in response to metaphors due to preceding contextual information (Pynte et al., 1996; Bambini et al., 2016) as well as cloze probability (Tartter et al., 2002).

The question whether cueing affects language comprehension has been investigated for non-verbal information including speech prosody (e.g., Morlec et al., 2001; Hellbernd and Sammler, 2016) and contextual constraints using emoticons (Thompson et al., 2016), knowledge about speakers (Lattner and Friederici, 2003; Van Berkum et al., 2008; Regel et al., 2010; Kuhlen et al., 2015), facial and gesture information (Cohn and Kutas, 2015; Obermeier et al., 2015). Speech prosody was shown to convey the speaker’s intention and to guide social communication as observed in speech act ratings (Hellbernd and Sammler, 2016). Such effects of prosody, however, were not seen during online comprehension of irony marked by a certain tone-of-voice (Regel et al., 2011). As the tone-of-voice accompanying irony varies across speakers (see also Bryant and Tree, 2005), more obvious and generally valid cues may be more effective in cueing ironic intentions. Emoticons, for example, applied to irony emphasized the speaker’s intention and modulated the emotional impact of those utterances (Thompson et al., 2016). Further, knowledge about a speaker’s communicative style immediately affected the comprehension of irony as indicated by modulation of P200 whenever the speaker’s style of communication was congruent with the established knowledge about this speaker (Regel et al., 2010). Similarly, information about a speaker’s gesture style was shown to influence the integration of gesture and speech (Obermeier et al., 2015). The observation of early effects of sentence context (e.g., speaker identity, cloze probability) (for review see Penolazzi et al., 2007; Hagoort, 2008; Van Berkum et al., 2008) implies that contextual constraints may already initially affect the sentence interpretation.

When and how language-accompanying cues that clarify a speaker’s intention affect language comprehension is still unresolved. In order to elucidate the interplay of verbal and cueing information, the present study examines this issue by application of punctuation (i.e., quotation marks). Previous studies showed that punctuation (i.e., commas) prevented from initial misinterpretations by resembling linguistic prosody, and thus seems to guide sentence parsing (Steinhauer, 2003; Drury et al., 2016). In the current experiment, critical words for sentence meanings were put in quotes (e.g., *“great”*), which was coherent to pragmatic conventions for irony, and incoherent for literal language (Gutzmann and Stei, 2011). Thus, the critical verbal and cueing information appeared at the same time. If communicative cueing has an impact on language comprehension, we hypothesize different ERP effects for cued and uncued sentence interpretations. For uncued irony the P200–P600 pattern should be replicated (Regel et al., 2011, 2014). For cued irony we predict a modulation of this pattern due to enriched contextual constraints (e.g., Hagoort, 2008; Van Berkum et al., 2008) affecting either initial, or later stages of processing: If cueing and verbal information interact initially, pragmatic reanalysis of non-literal meanings (indicated by P600) is expected to be initiated earlier than without cueing (i.e., before 500 ms post-stimulus onset). In case cueing effectively constrains ironic interpretations, early semantic processes might be redundant resulting in the reduction, or even the absence of P200. In addition, by attracting attention to the cued information, a P300 response associated with attention allocation (for review see Kok, 2001) might be elicited for cued compared to uncued stimuli. In contrast, if cueing and verbal information is processed in parallel, and cueing has an impact not until later stages of processing, a modulation of P600 is hypothesized with larger amplitude for cued than uncued irony. Such a finding would reflect processing costs for the integration of both types of information. Moreover, for cued literal sentences (e.g., *That’s “great”*) that comprise a pragmatic anomaly by incorrectly pointing to alternative interpretations, an enhanced P600 may emerge relative to uncued sentences (e.g., *That’s great*) indexing reanalysis of the ambiguous interpretation.

## Experiment 1

### Methods

#### Participants

Forty native German-speaking students [20 female, mean age 24.9 years (standard deviation (SD) 3.20)] from the University of Leipzig took part in the experiment. All subjects were right-handed, had normal or corrected-to-normal vision, and were paid for their participation. The study was conformed to the declaration of Helsinki. All participants gave signed informed consent in accordance with this declaration.

#### Stimulus Material

The stimuli consisted of 120 target sentences, whose meaning depended on the foregoing contextual information. In three to four context sentences depicting an everyday situation either a slightly disappointing event (e.g., a cup of coffee fell over), or a pleasant event (e.g., someone enjoyed a book) was described. The same target sentences (e.g., *That’s fantastic*) appeared with either events thereby achieving either an ironic, or a literal meaning. For each target sentence two types of contexts were created, thus resulting in a total of 240 short stories. The target sentence final word contained the critical information for potential sentence meanings. Samples of the stimuli are presented in Appendix A. For the cueing condition, half of the target sentences critical words included quotation marks. Thus, 50% of the items each were cued (e.g., *That’s “fantastic”*) and uncued (e.g., *That’s fantastic*). In case of irony, cueing was coherent to pragmatic conventions, whereas for literal language cueing was incoherent. Compared to the entire proportion of verbal information including context and target sentences, cued words were still less probable, and thus presumably more salient than uncued words.

Before the experiment, two pretests were conducted on the stimuli in order to assess semantic expectancy and interpretation of the target sentences. In the semantic expectancy test, 28 German-speaking students [12 female, mean age 24.0 years (SD 2.74)], and in the interpretation test 20 German-speaking students [10 female, mean age 23.9 years (SD 3.28)] participated. None of the participants of the pretests did take part in the EEG-Experiments 1 or 2. To test for semantic expectancy of the target sentence final word, a sentence completion task (Taylor, 1953) was performed. In this task, the sentence final word had to be completed with the most appropriate word for the respective sentential contexts. Sentences were chosen as experimental items, each time ironic and literal sentence completions were semantically related to the targets. Target sentence-final words showed an average cloze probability of 94.2% (SD 8.15). Ironic sentence completions were less expected (about 5%) than literal completions [paired *t*-test on items *t*(119) = 28.25, *p* < 0.0001]. In the interpretation test, target sentence meanings had to be rated on their degree of irony in the respective sentential contexts using a 5-point scale (1 = hardly ironic, 5 = highly ironic). Ironic sentences were rated with 4.3 (SD 0.37) and literal sentences with 1.5 (SD 0.49) confirming sentences’ different interpretations [paired *t*-test on items *t*(119) = 2187.9, *p* < 0.0001].

Experimental factors were *pragmatics* (ironic/literal) and *cueing* (cued/uncued), which were fully crossed yielding four experimental conditions. Stimuli (context and target sentences) were presented visually. The sentence final words subtended 2° to 4.8° of horizontal, and 1° of vertical visual angle. For cued words horizontal visual angle was 0.7° larger than for uncued words. For experimental presentation, the 120 items were equally divided into four lists (i.e., 30 items each) to avoid repetition of target sentences, and pseudorandomized. Each participant saw only one list.

#### Procedure

During the experimental session (about 50 min), participants were seated in a sound-attenuated cabin in front of a monitor (at a distance of about 100 cm). A trial started with the visual presentation of the context sentences in one block of three to four lines on the monitor in a self-paced reading mode (automatic continuation after 20 s). After presentation of a fixation cross for 200 ms, target sentences were presented word-by-word in rapid serial visual presentation with 300 ms per word and 200 ms in between. After sentence offset and a blank screen for 1500 ms, participants were presented with the experimental task (i.e., comprehension task, for examples see Appendix A). In this task, a test statement outlining prior contextual information had to be judged with a *yes* or *no* response (response time of maximum 6000 ms). The inter-trial interval was 1000 ms.

#### Data Recording and Analysis

Behavioral data contained the judgments on the comprehension task, and were analyzed in a repeated-measure ANOVA with the two-leveled factors *pragmatics* (ironic/literal) and *cueing* (cued/uncued) using *Statistical Analysis Software* (*SAS*, version 9.4). The electroencephalogram (EEG) was recorded continuously from 52 Ag/AgCl electrodes (i.e., Fp1, Fpz, Fp2, Af7, Af3, Afz, Af4, Af8, F7, F5, F3, Fz, F4, F6, F8, Ft7, Fc5, Fc3, Fcz, Fc4, Fc6, Ft8, T7, C5, C3, Cz, C4, C6, T8, Tp7, Cp5, Cp3, Cpz, Cp4, Cp6, Tp8, P7, P5, P3, Pz, P4, P6, P8, Po7, Po3, Poz, Po4, Po8, O1, Oz, O1 and left mastoid) mounted in an elastic cap (Electro Cap International). In order to control for eye movement artifacts, the bipolar horizontal and vertical electrooculogram (EOG) placed on the outer canthus of each eye was recorded. For continuous EEG and EOG recording a band pass (DC to 70 Hz) was used. The sampling rate was 250 Hz. EEG recordings were referenced online to the left mastoid, and re-referenced oﬄine to the average of the left and right mastoid. Electrode impedances were kept below 5 kΩ. For the ERP analysis, epochs of EEG data were averaged for the critical word for each electrode position for each experimental condition in the period of -200–1000 ms relative to stimulus onset of the critical word using *ERP Evaluation Package* (*EEP*, version 3.2.1). Only correctly answered trials free from any artifacts [∼10% rejections due to ocular or movement artifacts (EOG rejection ±40 μV)] entered the analysis. For cued items the percentage of rejections slightly differed from that for uncued language (i.e., to about 2%). For statistical analysis of the ERP data using *SAS* (version 9.4), three time windows were calculated according to our predictions of replicating previous findings of P200 and P600 effects (e.g., see Regel et al., 2011, 2014), as well as visual inspection of a potential P300 effect: *250–350 ms* (P200), *350–550 ms* (N400/P300), and *550–900 ms* (P600). ERPs were quantified using multivariate analyses of variance (MANOVA) to prevent violations of sphericity (Vasey and Thayer, 1987). For all time windows, an overall MANOVA was conducted on the mean amplitude values of all dependent variables and included the factors *pragmatics* (ironic/literal) and *cueing* (cued/uncued). For distributional ERP analysis, the topographic factors *anterior/posterior* (2) and *hemisphere* (left/right) were defined and clustered into four different *Regions of Interest* (ROIs) with each containing the mean values of seven electrodes: left anterior (Fp1, Af7, Af3, F5, F3, Ft7, Fc5, Fc3), left posterior (Cp5, Cp3, P5, P3, Po7, Po3, O1), right anterior (Fp2, Af4, Af8, F4, F6, Fc4, Fc6), and right posterior (Cp4, Cp6, P4, P6, P8, Po4, Po8, O2). Midline electrode positions (Fpz, Afz, Fz, Fcz, Cpz, Pz, Poz and Oz) were analyzed separately. Whenever the overall analysis showed significant interactions between the experimental and topographic factors, further analyses were carried out separately for the respective topographic factors. In case further interactions between the experimental factors were observed on a particular topographic factor level, subanalysis for the experimental factors were conducted. Similarly, whenever the midline analysis revealed significant interactions, the experimental factors were analyzed separately. Effects thereby obtained were corrected by the Bonferroni–Holm procedure. All effects revealing a significance level of *p* < 0.05, and *p* < 0.1 for marginal significance are reported.

### Results

#### Behavioral Data

For the judgments of the comprehension task, a mean accuracy rate of 95.3% (SD 3.80) was obtained indicating that participants performed excellently. The statistical analysis showed an interaction of pragmatics with cueing [*F*(1,39) = 7.59, *p* = 0.01]. Follow-up analyses for each sentence meaning separately showed a main effect of cueing [*F*(1,39) = 4.27, *p* = 0.04] for literal sentences only. Participants performed slightly better when literal sentences were uncued [mean accuracy rate 96.4% (SD 4.02)] than cued [mean accuracy rate 94.9% (SD 5.06)]. For comparision, the mean accuracy rates were 95.6% (SD 5.19) for cued irony, and 94.7% (SD 5.43) for uncued irony.

#### ERP Data

**FIGURE 1:**
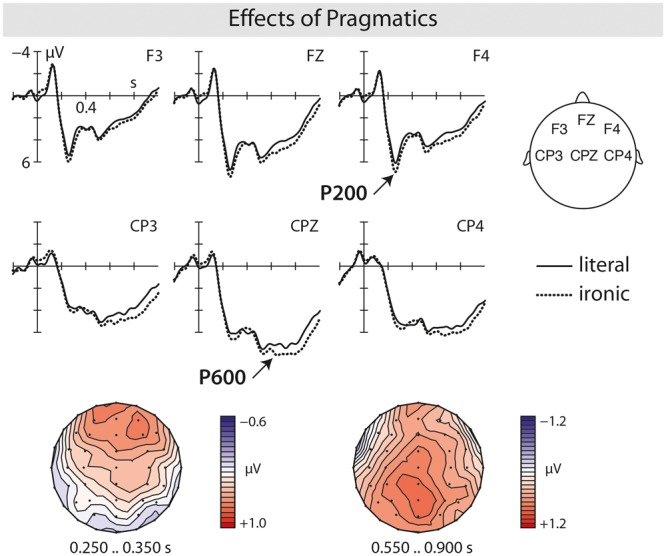
Grand average ERPs elicited by sentence final words achieving a literal meaning (solid line) and an ironic meaning (dotted line) with respect to the preceding context. For irony relative to literal language a P200–P600 pattern was elicited, in absence of an enhanced N400. The scalp distribution of the effects is shown by the topographic maps below. Note that the scaling of the topographic maps in all figures was adapted to the amplitude size of the effects.

**FIGURE 2:**
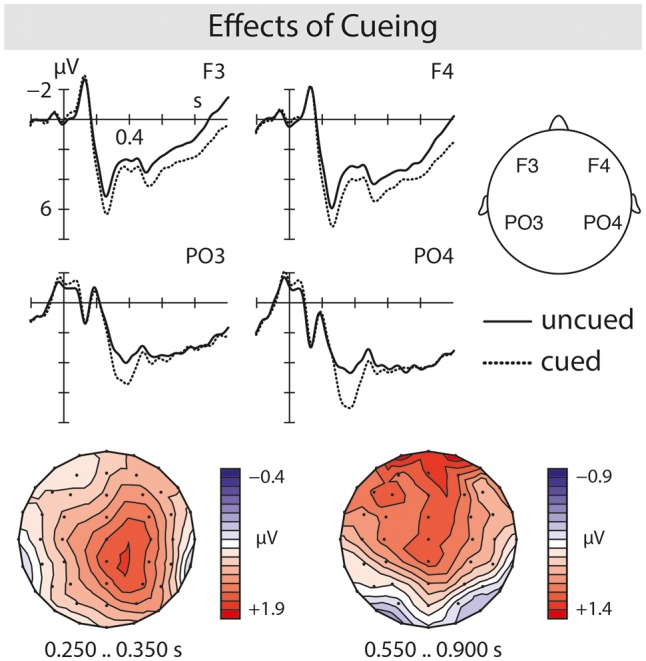
Grand average ERPs for cued (dotted line) compared to uncued (solid line) sentence final words revealed a P300 emerging around 250 ms post-stimulus presentation. Topographic maps of the P300 are shown below.

Grand average ERPs showed enhanced P200–P600 pattern for irony relative to literal language independent of cueing (**Figure 1**). An enhanced N400 amplitude related to irony was not seen. In response to cued sentences an enhanced positivity with a latency onset of around 250 ms was evoked compared to uncued ones (**Figure 2**).

The overall analysis of the *250–350 ms* time window revealed main effects of pragmatics [*F*(1,39) = 4.64, *p* = 0.04] and cueing [*F*(1,39) = 24.82, *p* < 0.0001], as well as a four-way interaction of pragmatics, cueing, anterior/posterior and hemisphere [*F*(1,39) = 4.12, *p* = 0.05]. Subanalysis for anterior and posterior electrode sites separately showed an anterior effect of pragmatics [*F*(1,39) = 9.05, *p* = 0.005] suggesting the emergence of a P200 effect for irony relative to literal language. Effects of cueing were observed anteriorly [*F*(1,39) = 14.85, *p* < 0.001] and posteriorly [*F*(1,39) = 24.41, *p* < 0.0001] indicating the presence of an enhanced early positivity resembling a P300 for cued compared to uncued sentences. Analysis of the midline electrodes showed main effects of pragmatics [*F*(1,39) = 4.39, *p* = 0.04] substantiating the P200 effect for irony, as well as of cueing [*F*(1,39) = 19.14, *p* < 0.0001] confirming an enhanced P300 for cued sentences.

In the *350–550 ms* time window, the overall analysis revealed a main effect of cueing [*F*(1,39) = 12.89, *p* < 0.001] suggesting that the P300 for cued relative to uncued sentences was still evident. Analysis of the midline electrodes confirms the presence of a P300 for cued sentences by showing an effect of cueing [*F*(1,39) = 11.34, *p* = 0.002]. Effects of pragmatics were not observed [*F*(1,39) < 0.53, *p* = 0.47] indicating that an enhanced irony-related N400 was not evoked.

In the main analysis of the *550–900 ms* time window, an effect of pragmatics [*F*(1,39) = 8.04, *p* = 0.007] and an interaction of pragmatics with anterior/posterior and hemisphere [*F*(1,39) = 4.79, *p* = 0.04] were found. The resolution of this interaction showed a significant effect for posterior electrode sites [*F*(1,39) = 10.05, *p* = 0.003] thereby confirming a parietally distributed P600 effect for irony compared to literal language. Further, in the main analysis an effect of cueing [*F*(1,39) = 6.88, *p* < 0.001], and an interaction of cueing with anterior/posterior [*F*(1,39) = 12.00, *p* = 0.002] were observed. By resolving this interaction by anterior and posterior sites, effects of cueing were obtained for anterior electrode sites only [*F*(1,39) = 18.38, *p* < 0.0001] indicating an enhanced predominantly anterior positivity for cued compared to uncued sentences. Analysis of the midline electrodes revealed a main effect of pragmatics [*F*(1,39) = 8.02, *p* = 0.007] substantiating the irony-related P600 effect in comparison to literal language. While an effect of cueing [*F*(1,39) = 8.50, *p* = 0.006] was also seen on midline electrodes, interactions of pragmatics with cueing were not present in this time window [*F*(1,39) < 0.11, *p* = 0.74].

### Discussion

This experiment investigated the impact of cueing communicative intentions on language comprehension. Critical words for potential sentence meanings were either cued by quotation marks, or uncued. For irony these cues pointed to the implied non-literal meanings, whereas for literal language those cues hindered appropriate meanings. Participants attentively processed the stimuli by showing an excellent performance on the comprehension task as confirmed by the behavioral data. Still, the incoherent cueing information negatively affected context judgments as seen for cued literal language. ERPs for irony compared to literal sentences revealed enhanced P200 and P600 effects, replicating the irony-related ERP pattern seen previously (Regel et al., 2010, 2011, 2014; Spotorno et al., 2013). An interaction of pragmatics and cueing, however, was not obtained (see also Supplementary Figure S1). The enhanced P200 in response to irony suggests initial semantic analysis processes (Federmeier et al., 2005; Wlotko and Federmeier, 2007) when encountering critical information in contexts biasing a non-literal sentence interpretation. While a semantic integration difficulty apparently not occurred as indicated by the absence of an enhanced N400 for irony relative to literal language, during later stages of processing pragmatic reanalysis seemed to be engaged as reflected by P600 (see e.g., Regel et al., 2014). Based on inferences on the message level speakers’ communicative intentions are derived (Grice, 1975; Giora, 2002). With regard to the impact of cueing communicative intentions, additional cues for speakers’ intentions did not affect the processing of sentence meanings, neither of coherently cued ironic meanings, nor of incoherently cued literal ones. As quotes accompanied both irony and literal language, this apparently caused an ambiguity in meaning of the cues thereby generally obscuring their function and preventing an impact on sentence interpretations. The absence of an interaction between the experimental factors *pragmatics* and *cueing* may have resulted from the applied design, in which both factors were fully crossed, and thereby asks for further investigation within an experimental design allowing an unambiguous interpretation of the cues. The observation of main effects of cueing suggests that this type of information has been processed in parallel to verbal information. For cued compared to uncued sentences a P300 response with a latency onset of around 250 ms emerged indicating enhanced processing related to punctuation. By cueing critical words and ascribing more salience to their meaning, the P300 might be a reflection of attention allocation towards those stimuli (see e.g., Kok, 2001). While the meaning of the quotations (i.e., cueing non-literal interpretation) seemed to be evaluated, this information was not integrated with the verbal information. The enhanced later anterior positivity for cued relative to uncued words might be associated with inhibition processes of the retrieved, but interpretation-irrelevant, information. The current finding contrasts punctuation by commas, which elicited a closure positive shift that has been associated with the processing of prosodic boundaries, and thus prevented from initial misinterpretations (Steinhauer, 2003; Drury et al., 2016). In the present study, quotes did not serve as a cue for sentence parsing, but may have been attended and evaluated with regard to their contextual enrichment. Alternatively, the present results might imply that semantic information outweighs the additional information of the cues when encountering the critical information for sentence interpretations. If this holds true, similar findings should be seen for unambiguous cueing of communicative intentions (i.e., when cues are coherently applied to pragmatic conventions). In order to disentangle whether an impact of cueing indeed depends on the cues’ meaningfulness, or may be rather absent in general due to the prevalence of semantic information needs further investigation by avoiding functional ambiguity of these cues. Thus, functionally unambiguous cues as occurring in natural language use are applied in Experiment 2 to assess their impact on sentence interpretations.

## Experiment 2

In case communicative cueing deviates from established pragmatic conventions (e.g., when occurring with literal language), this seems to override the function of those cues and prevents an integration of that information into language comprehension (see Experiment 1). For both cued ironic and literal intentions, cueing information appeared to be processed in parallel. Given the results of Experiment 1, we designed Experiment 2 to investigate whether an impact of cueing indeed depends on its functional unambiguity (i.e., by coherent use to pragmatic conventions), or rather is less relevant when encountering critical semantic information for particular sentence interpretations. Therefore, cueing of non-literal meanings (i.e., by quotation marks) was applied to irony only in coherence to pragmatic conventions (Gutzmann and Stei, 2011). Two separate experimental blocks allowed a comparison of the processing of cued and uncued language. In the first block, both irony and literal language were uncued, whereas in the second block only irony was cued. The same predictions hold as for Experiment 1. If cueing has an impact on language comprehension, then different ERP patterns should be seen for cued and uncued irony. In case verbal and cueing information is immediately integrated, the processing of cued and uncued irony is expected to diverge from initial stages of processing on, and pragmatic reanalysis engaged in irony comprehension might be initiated before 550 ms post-stimulus onset. If, however, cueing information is processed in parallel thereby not affecting language comprehension, interactions between cueing and irony should be absent. Such a finding would support the prevalence of semantic information for sentence interpretations. For uncued irony, a replication of the previously observed of P200-P600 pattern in absence of N400 is expected (Regel et al., 2011, 2014).

### Methods

#### Participants

Forty native German-speaking students [20 female, mean age 23.5 (SD 2.30)] from the University of Leipzig participated in the experiment, and were paid for their participation. All of them were right handed and had normal or corrected-to-normal vision. All participants gave signed informed consent in accordance with the declaration of Helsinki.

#### Stimulus Material and Procedure

The stimuli and the procedure were the same as in Experiment 1. Instead of applying cues to both irony and literal language, merely critical words of ironic sentences were put into quotations marks in the second block. In the first block, both literal and ironic sentences were uncued. Item lists were split into two blocks, so that each block contained a total of 60 items (i.e., 30 ironic and 30 literal sentences). Experimental factors were *pragmatics* (ironic/literal) and *cueing* (cued/uncued).

#### Data Acquisition and Analysis

The data acquisition and analysis was identical to Experiment 1. The statistical analyses included the factors *pragmatics* (ironic/literal) and *cueing* (cued/uncued). Rejections due to ocular or movement artifacts were comparable across conditions, in total about 12.5% of the trials. Due to larger visual angles for cued items slightly more trials (to about 3%) were rejected than for uncued items.

### Results

#### Behavioral Data

For the comprehension task, a mean accuracy rate of 96.2% (SD 3.01) was obtained suggesting an excellent performance of participants. The statistical analysis showed a significant effect of cueing [*F*(1,39) = 4.56, *p* < = 0.04] indicating that participants performed better in the first block including uncued sentences [mean accuracy 96.7% (SD 1.12)] than in the second block including cued and uncued sentences [mean accuracy 95.5% (SD 1.22)].

#### ERP Data

**FIGURE 3:**
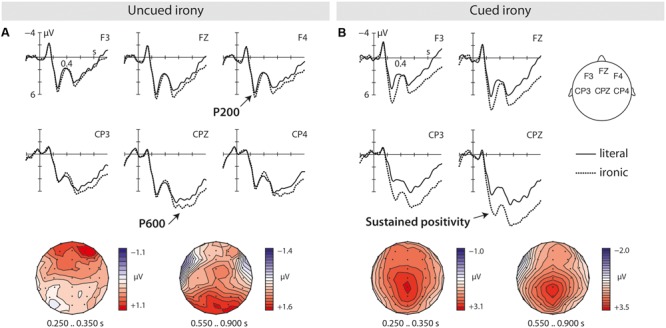
Grand average ERPs to literal (solid line) and ironic (dotted line) sentence final words shown for uncued irony **(A)** and cued irony **(B)** presented in two separate experimental blocks. Topographic maps of the ERP effects are displayed in the lower part of each column.

Grand average ERPs for uncued (**Figure 3A**) and cued irony (**Figure 3B**) revealed processing differences emerging already from 200 ms on. Compared to literal language uncued irony elicited a P200–P600 pattern, whereas for cued irony an enhanced P300 followed by a sustained positivity was evoked relative to uncued literal language.

The overall analyses of the *250–350 ms* time window revealed effects of pragmatics [*F*(1,39) = 26.59, *p* < 0.0001] and cueing [*F*(1,39) = 20.00, *p* < 0.0001], as well as a trend for a significant interaction between pragmatics, cueing and hemisphere [*F*(1,39) = 3.25, *p* = 0.08]. Resolving this interaction by hemisphere revealed for the left [*F*(1,39) = 10.61, *p* = 0.002] and the right hemisphere [*F*(1,39) = 7.61, *p* = 0.009] further interactions of pragmatics and cueing. Separate analyses for cued and uncued irony showed marginal significance for a right hemispheric effect of pragmatics [*F*(1,39) = 2.81, *p* = 0.1] for uncued irony suggesting a trend toward the emergence of a P200 effect for ironic compared to literal sentences. For cued irony, effects of pragmatics were obtained for both the left [*F*(1,39) = 28.83, *p* < 0.0001] and right [*F*(1,39) = 20.07, *p* < 0.0001] hemisphere indicating the presence of an enhanced early positivity in comparison to uncued literal sentences. This positivity resembled the P300 in response to cueing seen in Experiment 1. Analysis of the midline electrodes confirms the presence of this positivity for cued irony. Subanalysis of an interaction of pragmatics with cueing [*F*(1,39) = 10.13, *p* = 0.003] showed effects of pragmatics for cued irony [*F*(1,39) = 26.16, *p* < 0.0001].

In the *350–550 ms* time window, the overall analysis revealed effects of pragmatics [*F*(1,39) = 14.93, *p* < 0.001] and cueing [*F*(1,39) = 19.73, *p* < 0.0001], as well as an interaction of pragmatics, cueing and anterior/posterior [*F*(1,39) = 15.63, *p* < 0.001]. The resolution of this interaction by anterior/posterior showed for posterior electrode sites a further interaction of pragmatics and cueing [*F*(1,39) = 21.34, *p* < 0.0001]. Resolving this interaction by cueing revealed for cued irony an effect of pragmatics [*F*(1,39) = 29.98, *p* < 0.0001] indicating a sustained positivity effect compared to uncued literal sentences. Effects of pragmatics were not observed for uncued irony [*F*(1,39) = 0.00, *p* < 0.99] substantiating that an enhanced irony-related N400 was not elicited. In the statistical analysis of the midline electrodes, main effects of pragmatics [*F*(1,39) = 17.34, *p* < 0.001] and cueing [*F*(1,39) = 19.85, *p* < 0.0001], as well as an interaction of pragmatics and cueing [*F*(1,39) = 9.70, *p* = 0.004] were observed. The resolution of this interaction by cueing revealed an effect of pragmatics [*F*(1,39) = 21.09, *p* < 0.0001] merely for cued irony thereby confirming the presence of an enhanced sustained positivity effect for cued irony relative to literal language.

In the time window of *550–900 ms*, an effect of pragmatics [*F*(1,39) = 35.11, *p* < 0.0001] was found, as well as trends for an effect of cueing [*F*(1,39) = 2.95, *p* = 0.09] and an interaction of pragmatics and cueing [*F*(1,39) = 3.86, *p* = 0.06]. In separate analyses for cued and uncued irony, effects of pragmatics were seen for uncued irony [*F*(1,39) = 5.47, *p* = 0.03] suggesting the emergence of an enhanced irony-related P600 in comparison to literal sentences. For cued irony an effect of pragmatics [*F*(1,39) = 27.82, *p* < 0.0001] was also found confirming the enhanced sustained positivity for cued irony compared to uncued literal equivalents. Statistical analysis of the midline electrodes further substantiates both the findings by showing an interaction of pragmatics and cueing [*F*(1,39) = 4.27, *p* = 0.05]. In separate statistical analysis for cued and uncued irony, effects of pragmatics were significant for both cued [*F*(1,39) = 5.95, *p* = 0.02] (i.e., an enhanced sustained positivity) and uncued irony [*F*(1,39) = 34.05, *p* < 0.0001] (i.e., an enhanced P600) in comparison to literal language.

In order to analyze whether the sustained positivity effect seen for cued irony in Experiment 2 resulted from an additivity of two independent effects, namely the P300 effect of cueing plus the P600 effect of pragmatics related to irony, the difference waves of the main effect of cueing of Experiment 1 (i.e., cued vs. uncued language) and the effect for cued irony of Experiment 2 (i.e., cued irony vs. uncued literal language) were compared in the 550–900 ms time window. In case both the effects of cueing are generated by at least partially independent neural networks, distributional differences would be present in absence of an additive effect. Analysis of the difference waves included the factors *difference* (2), *anterior/posterior* (2) and *hemisphere* (2), and confirmed an overall significant difference between both the effects [*F*(1,39) = 9.61, *p* = 0.005]. This difference was substantiated on anterior [*F*(1,39) = 6.07, *p* = 0.02] and posterior electrode sites [*F*(1,39) = 8.30, *p* = 0.01] suggesting distributional differences between the effects for cueing and cued irony, and thus the absence of an additive effect on the observed sustained positivity in Experiment 2.

Further, to test whether this sustained positivity effect in response to cued irony differs from the P300 effect seen for cueing in Experiment 1, the difference waves of the two effects were also compared in the *250–350 ms* as well as the *350–550 ms* time windows. In the *250–350 ms* time window, a trend for an overall difference [*F*(1,39) = 2.95, *p* = 0.09] was observed. Analysis of the effects in the *350–550 ms* time window revealed an overall significant difference [*F*(1,39) = 11.53, *p* = 0.005] that was confirmed anteriorly [*F*(1,39) = 7.90, *p* = 0.01] as well as posteriorly [*F*(1,39) = 9.69, *p* = 0.005]. Both the P300 in response to cueing and the sustained positivity in response cued irony differed beginning at least from 350 ms post-stimulus onset on.

### Discussion

Experiment 2 investigated whether comprehending non-literal meanings benefits from cueing communicative intentions for irony (i.e., by quotation marks in the second block vs. no additional cueing in the first block), and scrutinized a potential prevalence of semantic information toward cueing information. Behaviorally, participants performed at ceiling in judging the context sentences, although performing slightly better in the first experimental block. In comparison to literal language, for cued and uncued irony a different pattern of results was seen: ERPs in response to cued irony revealed an enhanced P300 followed by a sustained positivity relative to literal equivalents, whereas for uncued irony compared to literal language the P600 was replicated, and a trend for a P200 effect was seen (Regel et al., 2010, 2011, 2014; Spotorno et al., 2013). As the current sustained positivity revealed a parietal topography in contrast to the more frontal main effect of cueing seen in Experiment 1, the current positivity may not reflect an additive effect of the irony- and cueing related positivity. The finding of different ERP patterns suggests the involvement of different processing mechanisms in presence of cueing by punctuation. The observed P300 followed by a sustained positivity for cued irony may be associated with the evaluation of cueing information and later integration of its meaning presumably allowing an immediate interpretation of the speaker’s intention based on that additional endorsement. The large amplitude of the sustained positivity suggests profound processing of communicative intentions by integrating this additional cueing information with the verbal input. In case sufficient evidence for a particular sentence interpretation is provided, processes of pragmatic reanalysis allowing a derivation of ironic intentions might be initiated immediately after allocating attention to the critical information. The observation of distributional differences between the P300 and later positivity for cueing seen in Experiment 1, and the current sustained positivity for cued irony suggests the engagement of different neural networks beginning from 350 ms on. Based on this finding, an alternative interpretation of the current effects for cued irony reflecting two independent consecutive processes (e.g., attention allocation as indexed by P300, and cueing-independent pragmatic revision as indexed by P600) appear to be less likely. The findings rather imply that when cueing is meaningful, this information immediately affects sentence interpretation. In contrast, when dealing with uncued irony speakers’ intentions are less evident and rely merely on verbal and contextual information engaging distinct comprehension processes: Early semantic analysis for retrieving extended semantic information of words as biased by the preceding context (indicated by the trend for an enhanced P200) precedes later pragmatic reanalysis of the speaker’s message (indicated by enhanced P600) allowing the derivation of the communicative intention. Moreover, the data imply that cueing intentions cannot evade enhanced pragmatic processing (i.e., presumably pragmatic reanalysis), as shown by the emergence of the sustained positivity for cued irony. Punctuation cues, thus, might not specify communicative intentions *per se*, but seem to emphasize respective interpretations along with verbal information. On basis of Experiments 1 and 2, an impact of cueing information on language comprehension seems to depend on its unambiguous usage according to pragmatic conventions. With regard to a potential prevalence of semantic information towards cueing information, the findings indicate that both types of information may be integrated immediately in case of unambiguous cueing. The present findings suggest that cueing information is apparently incorporated in language processing, rather than being processed in parallel, whenever it matches with pragmatic conventions.

## General Discussion

The role of cueing by prosody and emoticons for sentence interpretation was recently confirmed in behavioral studies (e.g., Hellbernd and Sammler, 2016; Thompson et al., 2016). The question whether cueing communicative intentions also affects online language comprehension, and whether such an impact occurs irrespective of the way of cueing examined by different experimental designs is still unresolved. Here, we investigated these issues by measuring ERPs for sentences cued by punctuation (Gutzmann and Stei, 2011) relative to uncued sentences. ERPs revealed that cueing by quotation marks can have an immediate effect on non-literal language comprehension, such as irony. For cued irony compared to literal language a P300 preceding a sustained positivity was obtained, whereas uncued irony elicited a trend for a P200 followed by a P600 effect (see Experiment 2). This sustained effect suggests the engagement of different neurocognitive processes for cued and uncued language comprehension. Besides verbal information processing, the more salient cueing information seems to be evaluated and integrated with semantic information. As cues provide sufficient evidence for non-literal interpretations, processes of pragmatic reanalysis might be preponed thereby enabling an immediate derivation of communicative intentions. The present data accord with findings of an effect of non-verbal information on language processing, either for disambiguating sentence meanings (Obermeier et al., 2015), or processing of visual narratives (Cohn and Kutas, 2015). Still, the different types of cueing may influence different stages of stimulus processing. While in the current study cueing seemed to affect stages of semantic and pragmatic processing (Experiment 2), gesturing merely bore on semantic processing (N400) in disambiguating speech (Obermeier et al., 2015), and subtle visual cueing by facial expression had an effect on revision processes of mental models (P600) (Cohn and Kutas, 2015). Moreover, communicative cueing depended on its usage according to established pragmatic conventions. Whenever cueing was incoherent with those pragmatic conventions in some cases (e.g., by applying quotations also to literal language), an influence of cueing language processing was not significant (Experiment 1) suggesting that their meaning was not relied on during sentence interpretation. Thus, methodological factors of the employed experimental designs apparently affected the way the cues were integrated into pragmatic processing of sentence interpretations. By including an incoherent cueing condition (as in Experiment 1), this might have affected the impact of the cues in the coherent condition. Irrespective of cueing, both experiments showed a P200–P600 pattern for irony compared to literal language thereby replicating previous findings (Regel et al., 2010, 2011, 2014; Spotorno et al., 2013). In absence of additional cues for speakers’ intentions, the processing of irony may engage initial processes of semantic analysis (P200), and later processes of pragmatic reanalysis allowing the derivation of intended sentence interpretations. Semantic integration difficulty (indicated by enhanced N400) of ironic word meanings, however, was not found.

With regard to neurocognitive approaches on non-literal language processing, the present findings partially accord with the assumptions of the *standard pragmatic view* (Grice, 1975). The observation of a P600 for irony supports the assumed later inferential processes, which, however, seems to occur independently of a prior semantic integration difficulty (indicated by the absence of an enhanced N400 amplitude in response to irony). Further, the emergence of P200 in response to irony suggests that initial stages of processing differed from that of literal language. In case sentential contexts are enriched by cueing information for the processing of irony a P300 and a sustained positivity was evoked implying the integration of additional information during interpretation of intentions, as well as an apparently earlier initiation of those processes than in absence of cues. Regarding initial stages of processing the present data ask for an adaption of the *standard pragmatic model* concerning early contextual effects. By obtaining early (P200) and late (P600) ERP effects a direct comprehension of non-literal sentence meanings as assumed by the *direct access view* (Gibbs, 2002) cannot be supported. Yet, enriched sentential contexts (i.e., by cueing relevant communicative intentions) did not allow for a direct understanding, but showed a modulation of the processing mechanisms engaged in comprehending speakers’ messages. While the present data suggests that cueing communicative intentions by punctuation affects language comprehension at various stages of processing, further investigation is required with respect to other types of cues, such as prosody, gestures, or emoticons, whether those cues are functionally comparable and imply more general principles.

## Conclusion

The role of cueing for interpreting communicative intentions was investigated in two experiments by applying punctuation (i.e., quotations) to irony (in Experiments 1 and 2) and literal language (in Experiment 1). The current findings indicate that cueing communicative intentions can have an immediate impact on language comprehension, given that the way of cueing accords with pragmatic conventions and methodological factors, such as the experimental design, would not impede its function. In this case, the presence of cues modulates the processing mechanisms underlying non-literal language comprehension: Whereas in the absence of cues initial semantic processes precede pragmatic reanalysis (as shown by an enhanced P200–P600 for irony relative to literal language), in the presence of cues attention is allocated to the cued information (as indexed by P300), which seems to be immediately integrated during pragmatic reanalysis of sentence interpretations (as shown by sustained positivity effect for cued irony compared to uncued literal equivalents). In the case cueing deviates from pragmatic conventions, however, cueing information seems to be processed in parallel to verbal information, and is not relied on for deriving communicative intentions.

## Ethics Statement

Prior to the experiments, all participants gave signed informed consent in accordance with the declaration of Helsinki. The study was approved by the ethics committee of the medical department at the University of Leipzig.

## Author Contributions

SR conceptualized the design and stimuli, undertook the acquisition, analysis, and interpretation of the data, and drafted the manuscript. TG substantially contributed to the conception of the design as well as the interpretation of the findings, critically revised and finally approved the to-be-published version of the manuscript.

## Conflict of Interest Statement

The authors declare that the research was conducted in the absence of any commercial or financial relationships that could be construed as a potential conflict of interest.

## Supplementary Material

FIGURE S1 Grand average ERPs to literal (black line) and ironic (blue line) sentence final words that were either cued (dotted line) or uncued (solid line).
