# Supplementary figures and images for: Don’t Get Me Wrong: ERP Evidence from Cueing Communicative Intentions
Source: Front Psychol. 2017 Sep 11;8:1465. doi: 10.3389/fpsyg.2017.01465 (PMC5600996; doi:10.3389/fpsyg.2017.01465)

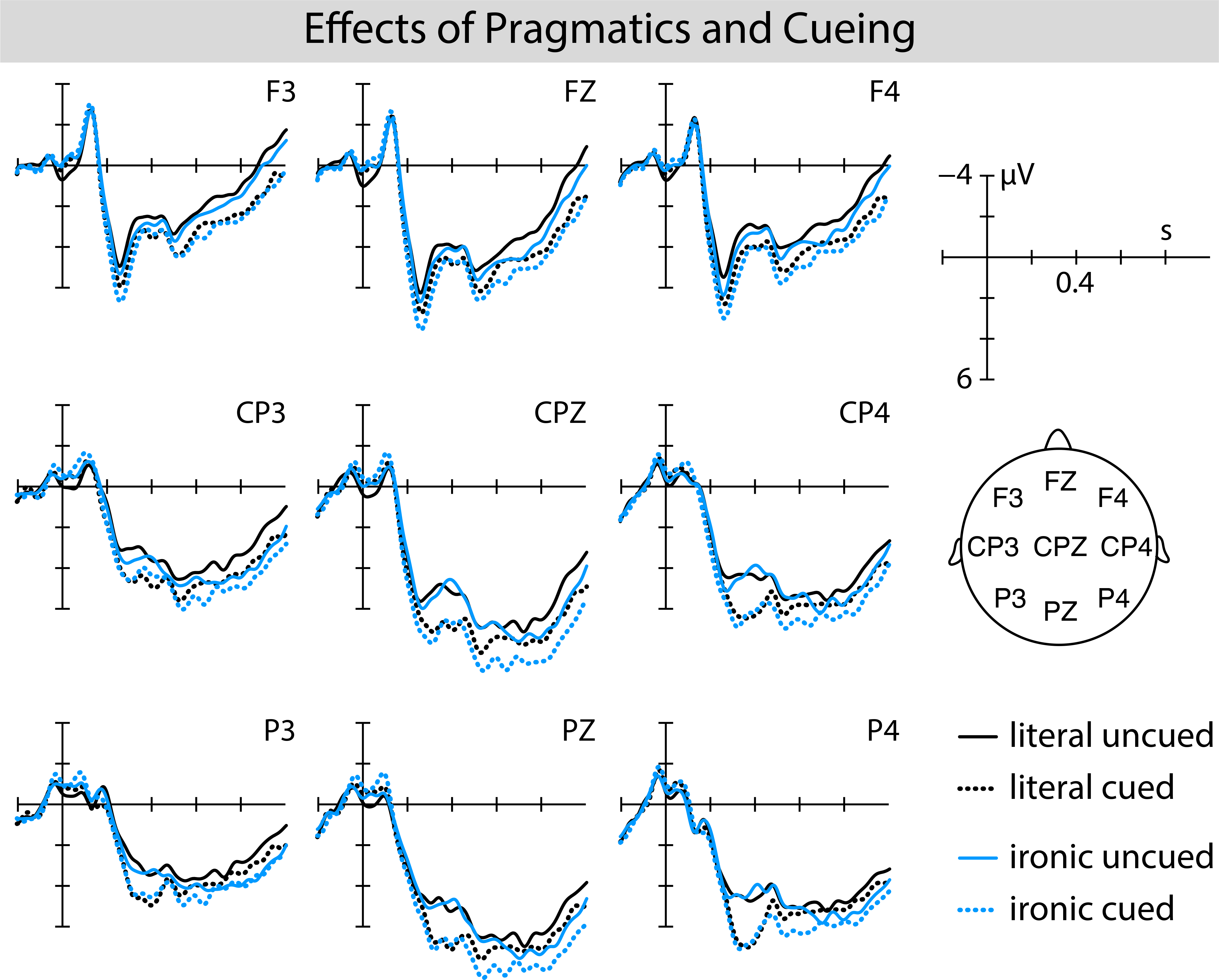

Supplement: FIGURE S1 — Grand average ERPs to literal (black line) and ironic (blue line) sentence final words that were either cued (dotted line) or uncued (solid line). [file Image_1.tif]
