# Supplementary material for: Don’t Get Me Wrong: ERP Evidence from Cueing Communicative Intentions
Source: Front Psychol. 2017 Sep 11;8:1465. doi: 10.3389/fpsyg.2017.01465 (PMC5600996; doi:10.3389/fpsyg.2017.01465)
Supplement: Supplementary file 2 [file Appendix_A.DOC]

**Appendix**. Samples of ironic stimuli (a) and literal stimuli (b) including examples of the test statements as applied in the comprehension task.

|  | **Contextual information** | **Target sentence** (uncued/cued) | **Test statement** |
| --- | --- | --- | --- |
| 1a | Sarah erklärt ihrem Kollegen, wie wichtig es wäre sich ausgewogen zu ernähren. Beim gemeinsamen Mittagessen entscheidet sie sich für einen Hamburger mit Pommes frites. Schmunzelnd bemerkt ihr Kollege: | Du ernährst dich ja wirklich gesund/“gesund“ | Sarah ernährt sich stets vorbildlich. |
| 1b | Sarah achtet immer sehr auf eine gute Ernährung. Sie isst viel Joghurt, Ballaststoffe und reichlich Obst. Beim Brunch mit einer Bekannten fällt dieser Sarahs Vollwertkost auf. Die Müslischale betrachtend sagt sie: |  | Sarah ernährt sich ausgewogen. |
| 2a | Stephanies neues WG-Zimmer führt zum Hinterhof hin. Der Blick aus dem Fenster zeigt ein altes baufälliges Mietshaus. Im Hof liegt dazu noch Schutt und Geröll. Als ihre Eltern sie besuchen und aus dem Fenster schauen, meinen sie: | Der Blick ist ja großartig/“großartig“ | Das Fenster zeigt einen Garten. |
| 2b | In Ulm wollte Claas zu gern den Kirchturm des Münsters besteigen. Mehr als 600 Stufen musste er hochgehen. Endlich oben angekommen, gab es eine herrliche Aussicht und Claas sagte begeistert: |  | Claas ist auf den Kirchturm gestiegen. |
| 3a | Auf der Tagung hält Professor Schmidt eine komplizierte Rede. Er macht viele Gedanken-sprünge, so dass man nur schwerlich folgen kann. Ein Teilnehmer hat es schon längst aufgegeben und murmelt am Ende verärgert: | Das war wirklich sehr aufschlussreich/ „aufschlussreich“ | Der Vortrag war unstruktiert. |
| 3b | Annelie und Kristina hören einen Vortrag über die Veränderung des Klimas. Interessiert folgen sie den detaillierten Ausführungen des Referenten. Am Ende sagt Annelie beeindruckt: |  | Annelie fand den Vortrag langweilig. |
| 4a | Annelie und Simone wollen zusammen kochen. Annelie soll die Kartoffeln schälen. Das tut sie auch sehr sorgfältig, aber redet die meiste Zeit über. Als Simone wieder zu ihr hinsieht, hat sie erst zwei geschält. Entgeistert meint sie: | Du bist ja schon fertig/  „fertig“ | Simone wartet auf die Kartoffeln. |
| 4b | Die kleine Amelie hat ihre Aufgaben immer schnell erledigt. Selbst beim Basteln hat sie den komplizierten Kranich rasch zusammengefaltet. Die Betreuerin sieht auf den Vogel und sagt überrascht: |  | Amelie ist meist schnell fertig. |
| 5a | Herr Wagner möchte gern seine Hauptwohnung ummelden. Auf der Behörde wird er gebeten zwei Anträge auszufüllen. Als er sie abgibt, fehlt ihm ein weiteres Dokument. Den Papierkram in der Hand meint Herr Wagner verdutzt: | Das geht ja alles problemlos/“problemlos“ | Die Ummeldung ist sehr aufwendig. |
| 5b | Als Ulli und Dana nach Bulgarien fahren, haben sie große Bedenken wegen der Einreise. An der Grenze müssen sie lediglich ihre Pässe zeigen und können gleich weiter fahren. Überrascht meint Ulli: |  | Die Einreise war kompliziert. |
| APPROXIMATE TRANSLATION OF THE STIMULI FROM GERMAN | | | |
| 1a | Sarah tells a colleague that she is on a balanced diet with aplenty of fresh fruits and vegetables. When having lunch together, she chooses an hamburger with a large portion of french fries. Her colleague beholds her dish and says grinning: | You eat absolutely healthy/”healthy” | Sarah eats always ideally. |
| 1b | Sarah strictly takes care for her well-balanced nutrition. She eats yoghurt, dietary fibers and fruits. When brunching with a friend, her friend notices her healthy meal. Looking at her muesli she says: |  | Sarah eats always balanced. |
| 2a | Stephanie rented a new room facing the yard. The view from the window shows an old derelict building. The yard is full of rubble and debris. When her parents come visiting her and look out of the window, they say: | The view is great/”great” | The window shows a garden. |
| 2b | When visiting Ulm, Claas decided to ascend the steeple of the minster with more than 600 steps. Finally arriving the top, he had an incredible view. Claas said delighted: |  | Claas ascended the minster steeple. |
| 3a | In the introductory seminar Professor Taylor gave a complicated talk with many mental leaps. Most of the students could not follow. One of the listener had already given up, and murmured annoyed: | This is so informative/  “informative” | The talk was unstructured. |
| 3b | At the Technology Institute Annelie listened to a talk about the latest developments. Interested in the topic, she followed all the statements. She found out many new things and, impressed, said: |  | Annelie was impressed by the talk. |
| 4a | Ann and Simona decided to cook dinner. Ann offered to peel the potatoes but was only talking. Simona, quite hungry by now, looked at her and saw only two peeled potatoes. Grumpily she says: | You are really quick/  “quick” | Simona is waiting for the potatoes. |
| 4b | Little Ann always quickly finishes her homework. Even when she is doing handicrafts, she easily folds a paper crane. As her aunt watched her folding the bird, she is astonished and says: |  | Amelie has often quickly finished. |
| 5a | Mr. Wagner needs to re-register at the record section. After he has arrived, he is asked to fill out two forms. When he hands in his documents, another form is still missing. With all the paperwork in his hands, he says irritated: | That is all that unproblematic /”unproblematic” | The notification was intricately. |
| 5b | Ulli and Dana are travelling to Bulgaria. At the frontier they have concerns about the entry. However, they only had to show their passports, and were allowed to continue their journey. Pleasantly surprised about it Ulli says: |  | The entry was complicated. |
